# Aortic arch type, a novel morphological indicator and the risk for acute type B aortic dissection

**DOI:** 10.1093/icvts/ivab359

**Published:** 2021-12-22

**Authors:** Likun Sun, Jiehua Li, Zhenyu Liu, Quanming Li, Hao He, Xin Li, Ming Li, Tun Wang, Lunchang Wang, Yuan Peng, Hui Wang, Chang Shu

**Affiliations:** 1 Department of Vascular Surgery, The Second Xiangya Hospital of Central South University, Changsha, China; 2 Vascular Disease Institute of Central South University, Changsha, China; 3 Department of Vascular Surgery, Fuwai Hospital, Chinese Academy of Medical Sciences & Peking Union Medical College, Beijing, China

**Keywords:** Aortic arch type, Type III arch, Morphological feature, Acute type B aortic dissection

## Abstract

**OBJECTIVES:**

Aortic arch type is a readily recognizable and obtainable morphological feature of the aorta that does not require complex measurements. The goal of this study was to evaluate whether aortic arch type is a comparable and alternative morphological parameter for predicting acute type B aortic dissection (aTBAD) by comparing the prognostic value of the aortic arch type with that of other morphological parameters such as aortic length, angulation and tortuosity index.

**METHODS:**

The patients with aTBAD (*n* = 216) were matched 1:1 with a control group (*n* = 263) by propensity score matching. The morphological data of the ascending aorta and the aortic arch, which included diameter, length, angulation and tortuosity index, were collected on a three-dimensional aortic model using 3mensio Vascular software. The aortic arch type was identified by the vertical distance from the origin of the brachiocephalic trunk to the top of the arch. The binary logistic regression models were analysed to determine the independent geometric variables related to the aTBAD. The nonparametric approach was performed to assess whether there were statistical differences between the area under the receiver operating characteristic curves (AUC) of the models.

**RESULTS:**

After propensity score matching, 151 matched pairs of patients were selected. The diameters at the sinotubular junction and the mid-ascending aorta, the ascending aorta length and the ascending aorta angulation in the aTBAD group were significantly greater than those of the controls. Compared with the control group, the diameters at the proximal aortic arch, mid-aortic arch and distal aortic arch, the angulation and the tortuosity index of the aortic arch were significantly greater in the aTBAD group. The proportion of the type III arch in the patients with aTBAD is higher than that of the type I arch and the type II arch (*χ*^2^ = 70.187; *P *<* *0.001). Binary logistic regression analysis showed that the diameter at the mid-aortic arch, the ascending aorta length, the aortic arch angulation and the tortuosity index were independently related to the aTBAD with an AUC value of 0.887. Another binary logistic regression analysis indicated that the diameter at the mid-aortic arch and the aortic arch type were independent correlative variables associated with the aTBAD with an AUC of 0.874. No significant difference was observed in the prognostic value of receiver operating characteristic curves between the 2 models (*P *=* *0.716).

**CONCLUSIONS:**

The type III arch, which has the characteristics of aortic elongation, incremental angulation and tortuosity index, is a comparable and alternative identifier for patients at high risk for aTBAD.

**Figure:**
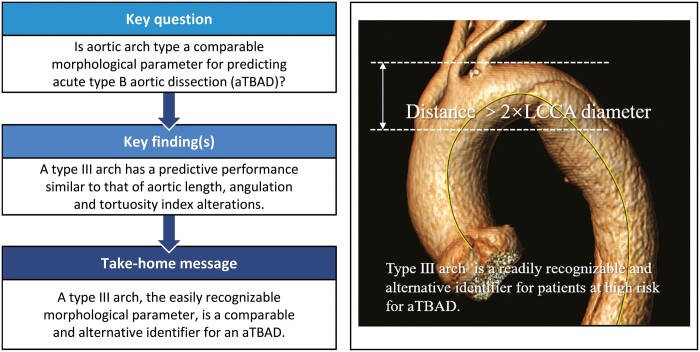

## INTRODUCTION

Acute type B aortic dissection (aTBAD) is an aortic disease that occurs in the acute phase caused by a tear in the intimal layer of the aorta distal to the left subclavian artery, with subsequent separation of the media from the intimal layer, that allows blood to flow into the newly formed false lumen [1]. Its diagnosis can be easily ignored or delayed unless one sees the classical clinical manifestation, i.e. abrupt onset of severe pain in the chest, back or abdomen [2].

A previous publication deemed that the descending aortic diameter is an unsatisfactory parameter to prevent onset of the aTBAD and that other indicators are still needed to identify patients at risk for an aTBAD [3]. Recent publications have focused on new morphological parameters, including aortic length, angulation and tortuosity index in order to improve the efficiency of screening and facilitate early intervention in high-risk populations. The researchers found that elongation of aortic length, increase of aortic angulation and the tortuosity index were related to the occurrence of aTBAD, which may be an effective predictor of aTBAD [4–6]. However, these morphological parameters, which represent the spatial geometry of the aorta, cannot be measured on conventional computed tomography angiography two-dimensional images. Instead, vascular three-dimensional reconstruction software is required for spatial measurement, but it makes the risk assessment of aTBAD sophisticated and time-consuming. Moreover, the main issue concerning the predictive value of aortic morphological features is the lack of methodological consistency in the definition of such features in extant studies, which makes it difficult to incorporate the method into routine clinical practice.

Aortic arch type, a readily recognizable morphological feature of the aorta without complex measurements, was initially proposed to help determine the difficulty of inserting the carotid stent [7]. A type III arch possesses a typical shape, with the top of the arch located at the distal end of the supra-aortic branches, which has been reported to be related to the occurrence of type B aortic dissection [8]. As suggested by a previous study [8], the type III arch may be associated with increased angulation and tortuosity index and the elongation of the aorta; however, this has not been sufficiently demonstrated. In addition, no articles published thus far report whether the type III arch configuration can be used as a morphological risk factor for predicting an aTBAD.

The purpose of the study was to evaluate whether aortic arch type, as an easily recognizable morphological parameter, is a comparable and alternative risk factor for predicting aTBAD by comparing the prognostic value of a type III arch with other morphological parameters such as aortic length, angulation and tortuosity index.

## METHODS

### Ethics statement

The study was approved by the medical ethics committee of Xiangya Second Hospital of Central South University. We have registered the study (No. 2018S053) and followed the Declaration of Helsinki. Written informed consent was unnecessary due to the observational and retrospective nature of the study and the anonymity of patients.

### Study design

We retrospectively analysed the clinical and imaging data of the patients diagnosed with an aTBAD from May 2017 to December 2018. The patients with type B aortic dissection in the acute period (≤14 days) were included in the study group. From May 2016 to December 2018, patients diagnosed with non-aortic disease by computed tomography angiography examination in our centre served as the control group. The patients with connective tissue disease, a bicuspid aortic valve, traumatic dissection, non-A non-B aortic dissection, isolated abdominal aortic dissection, history of aortic open or endovascular surgery and arch branching variants in the 2 study groups were excluded. We planned to perform the propensity score matching (PSM) to reduce the confounding factors generated by the baseline demographics and cardiovascular risk factors.

### Image post-processing

**Figure 1:**
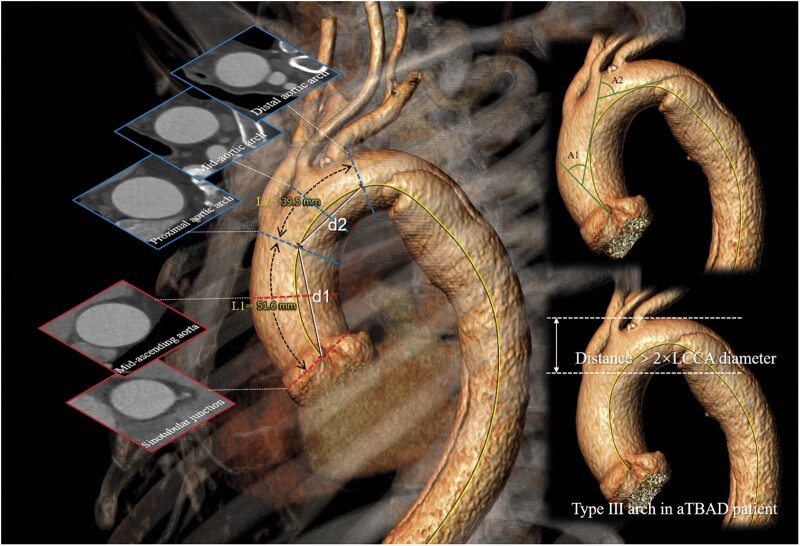
Measurement of morphological parameters of the ascending aorta and the aortic arch. The average aortic diameters were measured based on the areas of the planes perpendicular to the centreline. The red planes indicate the diameters of the sinotubular junction and mid-ascending aorta in the ascending aorta. The blue planes indicate the diameters of the proximal aortic arch, mid-aortic arch and distal aortic arch in the aortic arch. The ascending aorta length (L1) is the length of the centreline from the sinotubular junction plane to the proximal aortic arch plane (yellow line). The aortic arch length (L2) is the length of the centreline from the proximal aortic arch plane to the distal aortic arch plane (yellow line). Aortic angulations were measured by drawing tangent lines along the proximal and distal points of the centreline in the ascending aorta and aortic arch [9] (upper right panel: A1: ascending aorta angulation; A2: aortic arch angulation). Aortic tortuosity indexes were calculated as the ratio of the aortic length to the shortest linear distance between the beginning and end points in the ascending aorta and the aortic arch [10] (ascending aorta tortuosity index: T1 = L1/d1; aortic arch tortuosity index: T2 = L2/d2). Aortic arch type was identified by the vertical distance from the origin of the brachiocephalic trunk to the top of the arch. The distance of the type III arch was more than 2 times the diameter of the left common carotid artery in a patient with an acute type B aortic dissection (bottom right panel).

The Digital Imaging and Communications in Medicine standard data of the patients were post-processed using 3mensio Vascular software (version 10.0, The Netherlands). A centreline was created from the sinotubular junction to the aortic bifurcation. The total aorta was divided into different portions by the planes perpendicular to the centreline (Fig. 1). The diameters, lengths, angulations [9] and tortuosity indexes [10] of the ascending aorta and aortic arch were measured at various spatial planes (Fig. 1). According to the results of the previous study [7], the aortic arch type was determined by the vertical distance from the origin of the brachiocephalic trunk to the top of the arch. If the aortic dissection extends to the top of the arch, which interferes with the determination of the arch top, the level of the left subclavian artery can be conservatively regarded as the top of the arch [11]. The distance in a type I arch is <1 × left common carotid artery (LCCA) diameter; it is 1 × LCCA diameter ≤ the distance in a type II arch ≤2 × LCCA diameter; the distance in a type III arch >2 × LCCA diameter [7].

### Statistical methods

Continuous data were presented as mean ± standard deviation; categorical data were presented as numbers and percentages. The normality of the data was assessed by the Shapiro–Wilk test, histograms and standardized normal probability (*P*–*P*) plots. The Student's *t*-test and the Mann–Whitney test were used to compare continuous variables between the study groups. The χ^2^ test and the Fisher exact test were used to compare the categorical variables.

To reduce the confounding factors generated by the baseline demographics and cardiovascular risk factors, we used the PSM to further compare the differences between the 2 groups. The patients in the aTBAD group and in the control group were matched 1:1 using nearest-neighbour matching with replacement. The matching caliper was set at 0.05. Geometric variables associated with the aTBAD screening in univariable logistic regression models (*P* < 0.20) were included in the multivariable logistic regression models. The results were expressed as odds ratios with 95% confidence intervals (CIs). The receiver operating characteristic (ROC) curve and the area under the ROC curve (AUC) were used to assess the prognostic value of the models Statistical differences between the AUCs of the models were compared using the nonparametric approach [12]. Statistical analyses were conducted with SPSS software (version 22, IBM, Armonk, NY, USA) and MedCalc software (version 20, Ostend, Belgium). The *P*-values were calculated as two-sided *P*-values.

## RESULTS

### Baseline demographics and cardiovascular risk factors

The age, sex, body surface area, body mass index, current smoking status, peripheral artery disease and hypertension between the aTBAD group and the control group differed before the PSM. After the PSM, 151 pairs of patients were matched, and there were no significant differences in baseline demographics and cardiovascular risk factors between them (Table 1).

**Table 1:** Baseline demographics and cardiovascular risk factors in unmatched and matched cohorts

	Unmatched cohort				Matched cohort			
	aTBAD (*n* = 216)	Controls (*n* = 263)	Standardized difference^a^	*P*-Value	aTBAD (*n* = 151)	Controls (*n* = 151)	Standardized difference^a^	*P*-Value
Demographics								
Age, years	62.3 ± 10.2	56.2 ± 10.7	0.532	<0.001	58.5 ± 9.6	57.3 ± 11.2	0.083	0.32
Male	176 (81.5)	163 (62.0)	0.651	<0.001	127 (84.1)	124 (82.1)	0.026	0.65
BSA, m^2^	2.2 ± 0.5	1.8 ± 0.4	0.270	<0.001	1.9 ± 0.3	1.9 ± 0.3	−0.057	1.00
BMI, kg/m^2^	27.6 ± 3.5	26.9 ± 3.4	0.031	0.03	26.6 ± 3.4	26.3 ± 3.2	0.013	0.43
Cardiovascular risk factors								
Current smoker	139 (64.4)	142 (54.0)	−0.462	0.02	101 (66.9)	104 (68.9)	−0.085	0.71
Peripheral artery disease	92 (42.6)	83 (31.6)	0.124	0.01	78 (51.7)	75 (49.7)	0.096	0.73
Hypertension	169 (78.2)	160 (60.8)	0.125	<0.001	109 (72.2)	112 (74.2)	−0.058	0.70
Hyperlipidaemia	142 (65.7)	167 (63.5)	−0.354	0.61	107 (70.9)	101 (66.9)	0.006	0.46
Diabetes mellitus	68 (31.5)	65 (24.7)	0.153	0.10	52 (34.4)	47 (31.1)	0.045	0.54

Data are presented as mean ± standard deviation (SD) or *n* (%).

a Standardized difference is the ratio of the mean difference to the pooled standard deviation.

aTBAD: acute type B aortic dissection; BMI: body mass index; BSA: body surface area; SD: standard deviation.

### Geometric features in ascending aorta

The diameters at the sinotubular junction and mid-ascending aorta in the aTBAD group were significantly greater than those of the controls (all *P *<* *0.001), which were all within the physiological range. The length of the ascending aorta was significantly elongated in the group with aTBAD (*P *<* *0.001). The angulation of the ascending aorta in the aTBAD group was significantly greater than that of the control group (*P *=* *0.017; Table 2).

**Table 2:** Comparison of geometric parameters of the aorta in matched cohorts

	aTBAD (*n* = 151)	Control (*n* = 151)	*P*-Value
Ascending aorta			
Diameter at sinotubular junction, mm	32.2 ± 3.6	28.4 ± 3.1	<0.001
Diameter at mid-ascending aorta, mm	36.0 ± 3.2	33.5 ± 3.3	<0.001
Length, mm	74.6 ± 10.5	66.3 ± 8.4	<0.001
Angulation, degrees	84.8 ± 13.7	81.3 ± 11.5	0.017
Tortuosity index, %	116.2 ± 7.3	114.8 ± 6.2	0.074
Aortic arch			
Diameter at proximal aortic arch, mm	34.1 ± 3.3	31.2 ± 2.5	<0.001
Diameter at mid-aortic arch, mm	29.7 ± 3.2	27.4 ± 2.3	<0.001
Diameter at distal aortic arch, mm	27.0 ± 3.3	24.4 ± 2.5	<0.001
Length, mm	38.2 ± 7.3	37.3 ± 5.8	0.237
Angulation, degrees	53.4 ± 12.2	41.5 ± 10.9	<0.001
Tortuosity index, %	107.6 ± 4.1	105.8 ± 4.5	<0.001

Data are presented as mean ± standard deviation (SD).

aTBAD: acute type B aortic dissection; SD: standard deviation.

### Geometric features in the aortic arch

The diameters at the proximal aortic arch, the mid-aortic arch and the distal aortic arch in the aTBAD group were significantly greater than those of the controls (all *P *<* *0.001), all of which were within the physiological range. Compared with those in the control group, the angulation and tortuosity index of the aortic arch in the aTBAD group were significantly greater (all *P *<* *0.001; Table 2).

### Prevalence of arch type

The patients with type I, type II and type III arches accounted for 11.9% (18/151), 19.9% (30/151) and 68.2% (103/151) in the aTBAD group, respectively; the respective proportions for the control group were 35.8% (54/151), 43.7% (66/151) and 20.5% (31/151). There were significant differences in type I, type II and type III arches between the aTBAD group and the control group. The proportion of type III arches in patients with aTBAD was higher than that of type I and type II arches (*χ*^2^ = 70.187; *P *<* *0.001; Table 3).

**Table 3:** Comparison of aortic arch type in matched cohort

Matched cohort	Aortic arch type, *n* (%)			*χ* ^2^	*P*-Value
	Type I	Type II	Type III		
aTBAD (*n* = 151)	18 (11.9)	30 (19.9)	103 (68.2)	70.187	<0.001
Control (*n* = 151)	54 (35.8)	66 (43.7)	31 (20.5)		
*P*-Value	<0.001	<0.001	<0.001		

aTBAD: acute type B aortic dissection.

### Comparison of the prognostic value of acute type B aortic dissection based on geometric features

**Figure 2:**
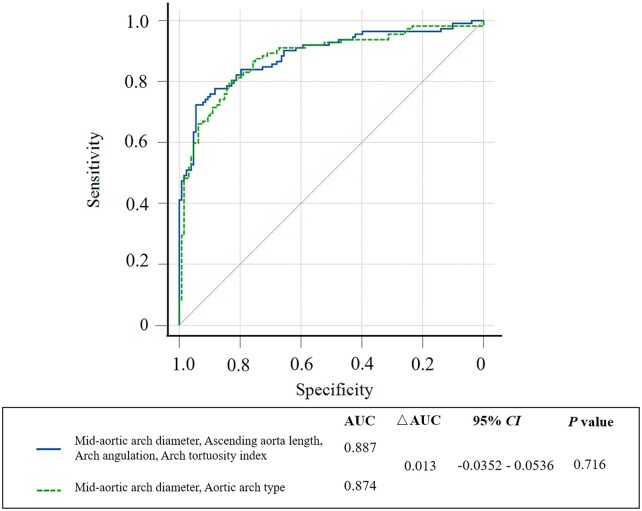
Comparison of the prognostic value of the receiver operating characteristic curves between the different models. The covariates of the binary logistic regression analysis (blue) included the diameters, lengths, angulations and tortuosity indexes of the ascending aorta and the aortic arch. The covariates of the binary logistic regression analysis (green) included the diameters of the ascending aorta, the aortic arch and the aortic arch type. The areas under the receiver operating characteristic curve of the 2 models were 0.887 and 0.874, respectively. No significant difference was observed in the prognostic value of receiver operating characteristic curves between the 2 models (*P *=* *0.716).

We included the diameters, lengths, angulations and tortuosity indexes of the ascending aorta and aortic arch as covariates in the binary logistic regression analysis and found that the diameter at the mid-aortic arch, the ascending aorta length, the aortic arch angulation and the aortic arch tortuosity index were independently related to the occurrence of aTBAD. The prognostic value of the model was significant with an AUC value of 0.887 (95% CI 0.846–0.927) (Table 4). Then, the diameters of the ascending aorta, the aortic arch and the aortic arch type were incorporated into the binary logistic regression analysis and showed that the diameter at the mid-aortic arch and the aortic arch type were independent correlative variables associated with the occurrence of aTBAD. The prognostic model yielded an AUC of 0.874 (95% CI 0.832–0.935) (Table 5). There was no significant difference in the predictive value of ROC curves between the 2 models (*P *=* *0.716) (Fig. 2).

**Table 4:** Binary logistic regression analysis for acute type B aortic dissection: length, angulation and tortuosity index

Variables	β coefficient	Standard error	Odds ratio	95% CI	*P*-Value	AUC
Diameter at mid-aortic arch	0.420	0.115	1.624	1.246, 1.873	<0.001	0.887
Ascending aorta length	0.368	0.124	1.405	1.146, 1.725	0.001	
Aortic arch angulation	0.121	0.043	1.126	1.045, 1.197	0.001	
Aortic arch tortuosity index	0.115	0.037	1.116	1.063, 1.189	<0.001	

Covariates of the analysis included the diameter, length, angulation and tortuosity index of the ascending aorta and aortic arch.

AUC: area under the receiver operating characteristic curve; CI: confidence interval.

**Table 5:** Binary logistic regression analysis for acute type B aortic dissection: arch type

Variables	β coefficient	Standard error	Odds ratio	95% CI	*P*-Value	AUC
Diameter at mid-aortic arch	0.432	0.105	1.532	1.228, 1.857	<0.001	0.874
Type III arch	0.113	0.034	1.124	1.053, 1.189	<0.001	

Covariates of the analysis included the diameter of the ascending aorta and aortic arch and arch type.

AUC: area under the receiver operating characteristic curve; CI: confidence interval.

## DISCUSSION

The association of aortic morphological features with aortic dissection has been the focus of intensive research over the years. Several published studies have demonstrated that aortic diameter, length, angulation and tortuosity index were associated with the occurrence of aTBAD [4–6], but the complex measurements of these parameters limit the improvement of the cost-effectiveness in screening programmes. The aortic arch type is a particular morphological parameter that can be easily recognizable and obtainable to reflect the shape of the aortic arch. We found that (i) ascending aortic length and aortic arch diameter, angulation and tortuosity index were independently related to the onset of aTBAD; (ii) aortic arch diameter and type III arch were independently associated with the onset of aTBAD; and (iii) no significant difference was noted between the prognostic value of a type III arch and the prognostic value of aortic length, angulation and tortuosity index in the development of aTBAD.

According to previous studies, the geometric changes of the dissected aortic segment are obvious, which is unsuitable for studying the morphological differences of the aorta before dissection [13, 14]. Therefore, our study only measured the morphological parameters of the aortic segment proximal to the orifice of the left subclavian artery that were not affected by dissection. The study showed that the diameters of the ascending aorta and aortic arch, the length of the ascending aorta, the angulations of the ascending aorta and the aortic arch and the arch tortuosity index in patients with aTBAD were significantly greater than those in the control group. Furthermore, binary logistic regression analysis indicated that ascending aortic length and aortic arch diameter, angulation and tortuosity index may play independent and specific roles in the development of an aTBAD. These findings are similar to those of previous studies [4, 6], suggesting that morphological changes in the ascending aorta and aortic arch may help identify patients at high risk of developing aTBAD.

However, the complexity and inconsistency of available measurement methods represent a clinical issue that requires a readily recognizable parameter to identify patients at high risk of aTBAD, for the sake of aggressive prophylaxis and treatment. To achieve this goal, we also compared the proportion of 3 types of aortic arches in the 2 groups and found that the proportion of type III arches in the aTBAD group was significantly higher than in the controls, which was consistent with the study results of Marrocco-Trischitta *et al.* [8]. These findings suggested that a type III arch may be an easily identifiable indicator of patients at high risk for aTBAD.

Three different methods for classifying aortic arch types are reported in contemporary publications and guidelines [15, 16] (proposed by Casserly [17], MacDonald *et al.* [18] and Madhwal *et al.* [7]), and each arch type can be divided into 3 types based on each classification method [19]. However, differences in the methodological approaches may lead to discrepancies in the arch type results [19, 20], which may overshadow the clinical relevance of the useful classification criterion and its predictive value for aTBAD. According to the classification method proposed by Casserly [17], the orifice of the brachiocephalic trunk is below the level of the inner curvature of the aortic arch in the type III arch. When the aortic dissection extends to the inner curvature of the aortic arch, the level mentioned previously will move downwards horizontally, which may bias the assessment of the aortic arch type. The classification method of MacDonald *et al.* [18] indicated that the vertical distance from the brachiocephalic trunk to the top of the aortic arch in the type III arch is >2 cm. However, it may be unreasonable to set the threshold of 2 cm as the classification criterion, because the aortic size is susceptible to individual height, weight and gender. In our study, we suggested that the classification method of Madhwal *et al.* could be a more suitable choice, because it is less affected by the aortic wall lesions and warrants sufficient repeatability based on the diameter of the left common carotid artery.

The AUC of the 2 binary regression models were 0.887 and 0.874 respectively, which showed no statistical significance in prognostic value, indicating that the type III arch had a predictive performance similar to that of the alteration of morphological parameters (namely, incremental length, angulation and tortuosity index) and was an alternative risk factor for predicting an aTBAD. Moreover, our findings also supported the suggestion proposed by in a previous study to a certain extent: a type III arch is characterized by aortic elongation and greater angulation and tortuosity index compared with type I and type II arches, which is associated with a high risk of an aTBAD. This relation may be due to the specificity of the anatomical position of the ascending aorta and aortic arch, which is limited by the heart, supra-aortic branches and descending aorta. When the aorta is elongated, the restricted aorta bends, contributing to the increase of the angulation and tortuosity index of the aorta [6]. Accordingly, this specific biomechanical interplay between elongation, angulation and the tortuosity index may account for the formation of the type III arch and facilitate the prognostic value of changes in geometric and anatomical configurations.

### Limitations

First, owing to the retrospective design of our study, the findings in the present study require further validation via prospective studies. Second, as with most retrospective studies, there was still selection bias, even though we used PSM. Third, the Digital Imaging and Communications in Medicine data used in the measurement were based on the non-electrocardiogram-gated scans, and the cardiac motion artefacts may affect the accuracy of the measurements.

## CONCLUSION

Aortic arch type is the readily recognizable morphological parameter without complex measurements. A type III arch, which has the characteristics of aortic elongation, incremental angulation and tortuosity index, is a comparable and alternative identifier for patients at high risk for aTBAD.

## Funding

This work was supported by the National Natural Science Foundation of China (grant number: 81870345, 81800400 and 81900423) and the Natural Science Foundation of Hunan Province (grant number: 2019JJ50851).

**Conflict of interest:** none declared.

## Data Availability Statement

All relevant data are within the manuscript and its supporting information files.

## Author contributions

**Likun Sun:** Conceptualization; Data curation; Investigation; Methodology; Software; Writing—original draft; Writing—review & editing. **Jiehua Li:** Data curation; Investigation. **Zhenyu Liu:** Data curation; Methodology; Software. **Quanming Li:** Data curation; Methodology. **Hao He:** Data curation; Formal analysis. **Xin Li:** Investigation; Methodology. **Ming Li:** Data curation; Methodology. **Tun Wang:** Data curation; Formal analysis. **Lunchang Wang:** Investigation; Methodology. **Yuan Peng:** Investigation; Software. **Hui Wang:** Investigation; Methodology. **Chang Shu:** Conceptualization; Formal analysis; Funding acquisition; Writing—review & editing.

## Reviewer information

Interactive CardioVascular and Thoracic Surgery thanks the anonymous reviewer(s) for their contribution to the peer review process of this article.

## ABBREVIATIONS

aTBAD: Acute type B aortic dissection
AUC: Area under the receiver operating characteristic curves
CIs: Confidence intervals
LCCA: Left common carotid artery
PSM: Propensity score matching
ROC: Receiver operating characteristic
